# Coupling of *kenaf* Biochar and Magnetic BiFeO_3_ onto Cross-Linked Chitosan for Enhancing Separation Performance and Cr(VI) Ions Removal Efficiency

**DOI:** 10.3390/ijerph17030788

**Published:** 2020-01-27

**Authors:** Daixi Zhou, Guangyu Xie, Xinjiang Hu, Xiaoxi Cai, Yunlin Zhao, Xi Hu, Qi Jin, Xiaohua Fu, Xiaofei Tan, Chong Liang, Kaiqi Lai, Hui Wang, Chunfang Tang

**Affiliations:** 1 College of Environmental Science and Engineering, Central South University of Forestry and Technology, Changsha 410004, China; daixizhou07@126.com (D.Z.); anyuxie@126.com (G.X.); T20031513@csuft.edu.cn (X.F.); 15964519539@163.com (C.L.); lkq1799689599@163.com (K.L.); wanghui@csuft.edu.cn (H.W.); T20051104@csuft.edu.cn (C.T.); 2 College of Art and Design, Hunan First Normal University, Changsha 410205, China; xiaoxi@hnu.edu.cn; 3 Faculty of Life Science and Technology, Central South University of Forestry and Technology, Changsha 410004, China; T20142201@csuft.edu.cn (Y.Z.); jq305745239@126.com (Q.J.); 4 College of Environmental Science and Engineering, Hunan University, Changsha 410082, China; tanxf@hnu.edu.cn

**Keywords:** Magnetic biochar, Chitosan, Cr(VI) decontamination, Glutaraldehyde-crosslinking, Adsorption, Fractional factor design

## Abstract

Cr(VI) contamination has posed great threat to both the ecosystem and human health for its carcinogenic and mutagenic nature. A highly effective adsorbent for the removal of Cr(VI) was prepared and its adsorption mechanism was thoroughly discussed in this study. In detail, magnetic BiFeO_3_ and *kenaf* biochar were loaded on cross-linked chitosan to obtain chitosan-*kenaf* biochar@BiFeO_3_ (CKB) for improving adsorption capacity towards Cr(VI). The adsorption process of Cr(VI) onto CKB was evaluated as a function of the pH, the existence of competing ions, the initial concentration of Cr(VI) and contact time. The results show that CKB exhibits the highest adsorption capacity under the optimal pH 2.0. The presence of competing ions such as Ca^2+^, NO_3_^−^, SO_4_^2−^, and Cl^−^ decreases the adsorption capacity; among them, Ca^2+^ and NO_3_^−^ show the greatest hindrance. By studying the effect of initial Cr(VI) concentration on the adsorption capacity, it was found that CKB in the solution was enough to remove Cr(VI) for all treatments (10–200 mg/L). The adsorption experimental data were well fitted with pseudo-first-order model, suggesting that chemisorption is not the dominant rate-limiting step. Freundlich isotherm model can better explain the adsorption process, indicating a non-ideal adsorption towards Cr(VI) on a heterogeneous surface of CKB. A 2^5-1^ Fractional Factorial Design (FFD) showed that pH and initial concentration of Cr(VI) have significant influence on Cr(VI) adsorption in our reaction system. In general, excellent adsorption efficiency of CKB indicates that it may be a good candidate for the remediation of Cr(VI)-contaminating wastewater.

## 1. Introduction

In recent years, industries such as mining, printing, dye manufacturing, metallurgy, and electroplating have caused excessive emissions of chromium into the environment [1]. Chromium exists mainly in two valence states of Cr(VI) and Cr(Ⅲ) in the industrial wastewaters and the toxicity of Cr(VI) is much higher than Cr(Ⅲ) due to its solubility, mobility, and carcinogenic and mutagenic nature [2]. In 2004, World Health Organization acclaimed that 50 µg/L is the maximum allowable limit for total chromium amounts in drinking water [3]. The presence of Cr(VI) in ecosystems has become a global environmental problem [2]. Non-degradable Cr(VI) has the tendency for bioaccumulation in the food chain, seriously threatening human health and aquatic living organisms and potentially disturbing the entire ecosystem [4]. Traditional methods towards Cr-containing wastewater include electrolytic reduction, chemical precipitation, ion exchange, electro-osmosis, photocatalysis, and adsorption [5]. Among these methods, adsorption is considered the method of choice because it possesses the advantage of simplicity of operator, lower cost, higher treatment efficiency, and better effects of Cr(VI) decontamination [6,7,8]. The selecting of an adsorbent is a determining factor for high-efficient adsorption. 

Biochar, a carbon-rich material obtained from biomass, with large specific surface area, porous structure, various functional groups, and mineral components [9], has been widely used as an adsorbent for pollutants removal in soil and water [10,11,12]. In addition, it is both cost-efficient and resource-saving to apply biochar mainly obtained from solid waste and agricultural biomass as adsorbent towards pollutants. The raw material of biochar in this work is *kenaf*, with large biomass production, takes merely less than 6 months to obtain available size (at maturity) for practical application [13]. In order to further improve the Cr(VI) decontamination efficiency of biochar, its particle size is minimized so as to increase its surface area exposed to Cr(VI), thereby enhancing Cr(VI) adsorption. This method, however, leads to the difficulty of the separation and recycle of adsorbents because it is difficult to separate small-size and low-density adsorbents from aquatic solutions via sedimentation, centrifugation, filtration or other traditional separation methods [14]. In this case, magnetic separation is more applicable, in which some magnetically responsive nanomaterials such as Fe_3_O_4_ [15], zero-valent iron and BiFeO_3_ are doped in adsorbents to achieve easy solid-liquid separation using a magnet. 

BiFeO_3_, a multiferroic material, exhibits both antiferromagnetic and ferromagnetic properties [16]. The coupling of BiFeO_3_ to *kenaf* biochar to form biochar-based magnetic adsorbents are easy to separate and recycle from solutions under external magnetic field. In addition, BiFeO_3_, with a proper band gap at ~2.2 eV, has photocatalytic capacity under visible-light irradiation of reducing Cr(VI) to low-toxicity Cr(III), which further promotes the removal of Cr(VI) from wastewaters. 

To couple biochar to BiFeO_3_ in a compact and stable way, the linear chain-like biopolymer chitosan is served as a carrier for them. Meanwhile, chitosan is an ideal scavenger for heavy metal ions such as Cr(VI) because -NH_2_ and -OH on its surface can act as chelating sites that capture metal ions [17,18]. However, as chitosan tend to be partially dissolved in low-pH environment, it is not suitable for treating acidic industrial wastewater [19]. Studies found that chemical modification of chitosan such as crosslinking can effectively overcome its problems of acid dissolution, low mechanical strength, and poor thermal stability of chitosan [19,20]. Thus, in this work, the *kenaf* biochar and BiFeO_3_ are loaded on cross-linked chitosan using glutaraldehyde to form a physiochemically stable, recyclable, and high-efficiency ternary adsorbent (CKB).

In this paper, CKB composite was prepared and characterized. The effects of environmental condition and physiochemical properties (i.e., pH value, temperature, initial concentration of Cr(VI), and foreign anions) on Cr(VI) adsorption were discussed. The adsorption process was investigated through adsorption kinetics, isotherms models, and intra-particle diffusion model. A 2^5-1^ Fractional Factorial Design (FFD) was performed in order to investigate individual and combined effects of five factors that may affect the removal efficiency of Cr(VI) in practical wastewater treatment (A: pH, B: temperature, C: initial concentration of Cr(VI), D: NaCl, E: KH_2_PO_4_). 

## 2. Materials and Methods

### 2.1. Materials

Ferric nitrate nonahydrate (Fe(NO_3_)_3_·9H_2_O AR grade), Bismuth nitrate pentahydrate (Bi(NO_3_)_3_·5H_2_O, AR grade), chitosan ((C_6_H_11_NO_4_)N, BR level), glutaraldehyde (C_5_H_8_O_2_, AR grade, 50% in H_2_O), potassium dichromate (K_2_Cr_2_O_7_, AR grade), 2-Methoxyethanol (HOCH_2_CH_2_OCH_3_, AR), and ethylene glycol ((CH_2_OH)_2_, AR grade) were all bought from Sinopharm Chemical Reagent Co., Ltd. Acetone (C_3_H_6_O, AR grade) was purchased from Choron Chemical Co., Ltd. Citric acid (C_6_H_8_O_7_, AR grade) was supplied by Shanghai Macklin Biochemical Technology Co., Ltd. Potassium dichromate (K_2_Cr_2_O_7_, AR grade) and Acetic acid (C_2_H_4_O_2_, AR grade) were produced by Tianjin Fengchuan Chemical Reagent Technologies Co., Ltd. ( Tianjin, China).

### 2.2. Synthesis of CKB

BiFeO_3_ was prepared using the sol-gel method [21]. Firstly, 0.08 mol of Fe(NO_3_)_3_ and Bi(NO_3_)_3_ were dissolved in 200 mL of 2-Methoxyethanol and 0.2 mL of 0.1 mol/L nitric acid in a 500 mL beaker. Then 0.08 mol of citric acid was dissolved in 100 mL of ethylene glycol. After magnetically stirring (300 rpm) the mixture of abovementioned solutions, the mixture was heated for 1 h at 60 °C and then for 10 h at 100 °C to obtain a light brown gel; then, the gel was heated for 30 min at 200 °C and for 2 h at 500 °C in a crucible (500 mL, contain >99% Al_2_O_3_) in a muffle furnace. The sample was cooled and ground to obtain BiFeO_3_ material. 

The *kenaf* biochar was obtained through thermal method [22]. First, *kenaf* stems were picked, washed, naturally dried, and dehydrated in an oven at 70 °C and ground to powder. Next, the *kenaf* powder was heated to 450 °C for 2 h at a quartz boat in a tube furnace until pyrolysis was complete. The powder was cooled and screened to obtain *kenaf* biochar.

Synthesis of CKB was achieved by crosslinking chitosan via glutaraldehyde [23]. First, chitosan (5 g) was magnetically stirred at 300 rpm and dissolved in 250 mL of 1% acetic acid in a 500 mL beaker. Then, the obtained BiFeO_3_ (1 g) and *kenaf* biochar (2 g) were magnetically stirred at 300 rpm and dissolved in the solution and 50 mL of 1% glutaraldehyde was then added and stirred at 50 °C until the gel was precipitated out. Next, we let the gel stand for 12 h at 25 °C for full reaction, and then 45 mL of 4% NaOH solution was added to the gel to adjust pH value to 9.0. Next, the gel was heated in a water bath for 2 h at 50 °C, and 10 mL of acetone was added. Finally, rinse the solution and remove the supernatant until the precipitate were neutral. The precipitate was dried and ground to obtain CKB. 

### 2.3. Characterization

The microscopic shape of the composite was characterized by a US QUANTA 250 field emission scanning electron microscopy instrument (FE-SEM; FEI, Hillsboro, OR, USA). The elements contained in samples were analyzed by GENESIS Energy Disperse Spectroscopy (EDS; EDAX, Philadelphia, PA, USA). The infrared spectroscopy was performed on a NICOLET 5700 Fourier infrared spectrometer (FT-IR; Thermo Nicolet Corporation, Madison, WI, USA). The thermogravimetry-differential thermal analysis curve was measured by an SDT Q600 synchrotron thermal analyzer (TG-DTA; TA Corporation, New Castle, Delaware., USA). The phase compositions of the composite were analyzed by an ESCALAB 250Xi X-ray photoelectron spectrometer (XPS; Thermo Fisher Scientific, Waltham, MA, USA) and a D/max-2500 X-ray diffraction (XRD; Rigaku, Japan). The magnetism of the composite was performed on a MPMS (SQUID) -XL-7 vibrating sample magnetometer (VSM; Quantum Design Instruments, San Diego, CA, USA). The zeta potentials were measured using a Zetasizer Nano SZ (ZEN3690, Malvern, UK).

### 2.4. Adsorption Experiments

#### 2.4.1. Batch Adsorption Experiments

The adsorption experiment was performed in a ZWY-2102C constant-temperature culture oscillator. For each experiment, 50 mL of Cr(VI) and 0.2 g of adsorbent were added in a 100 mL Erlenmeyer flask. 0.01 or 0.1 M of NaOH and HCl solutions were added to adjust desired pH values. Then, the Erlenmeyer flasks were shaken for 4 h at a rotation speed of 150 rpm at desired temperatures. The adsorbent was then removed from the solution using a permanent magnet (5 × 5 × 3 cm^3^, 300 mT). The amount of remaining Cr(VI) was measured by a UV spectrometer at 540 nm using the diphenylcarbazide method [24]. The adsorption capacity (*q*_e_, mg/g) and adsorption percentage (*E*_e_, %) were calculated via Equations (1) and (2):

(1) $${\;q}_{e}=\frac{\left(C_{0}{-C}_{e}\right)V}{W}$$

(2) $$E_{e}=\frac{\left(C_{0}{-C}_{e}\right)\times 100}{C_{0}}$$

where *C*_0_ (mg/L) and *C*_e_ (mg/L) are concentration of Cr(VI) at the beginning and equilibrium of adsorption, respectively; *V* (mL) is solution volume; *W* (g) is mass of the adsorbent.

#### 2.4.2. 2^5-1^ Fractional Factorial Design

The main and interactive effects of five experimental factors (A: pH, B: temperature, C: initial concentration of Cr(VI), D: NaCl, E: KH_2_PO_4_) on adsorption ability (*q*_e_) were investigated using a 2^5-1^ FFD. The experimental design matrix and results were depicted in Table S1. Design Expert 8.0.6 (Stat-Ease Inc., USA) and Minitab Release 16 (Minitab Inc., USA) were used for data analysis.

### 2.5. Adsorption Kinetics and Isotherm Models

#### 2.5.1. Adsorption Kinetics Models

Adsorption kinetics were investigated by nonlinear pseudo-first-order, pseudo-second-order, and intra-particle diffusion models, which refer to Equations (3)–(5) [25,26], respectively:

(3) $$q_{t}{=q}_{e,1}\left({1-e}^{-k_{1}t}\right)$$

(4) $$q_{t}=\frac{q_{e,2}{}^{2}k_{2}t}{{1+q}_{e,2}k_{2}t}$$

(5) $${\;q}_{t}{=k}_{p}t^{0.5}+C$$

where *q*_t_ (mg/g) is adsorption capacity at time *t* (min); *k*_1_, *k*_2_ (g/mg min) refer to adsorption rate constants for pseudo-first-order and pseudo-second-order models, respectively; *q*_e,1_, *q*_e,2_ (mg/g) represent adsorption capacity at equilibrium estimated by pseudo-first-order and pseudo-second-order model, respectively; *k*_p_ (mg/g min^0.5^) is an intraparticle diffusion rate constant; and *C* is the intercept.

#### 2.5.2. Adsorption Isotherm Models

Adsorption isotherm models such as Langmuir, Freundlich, and Temkin are delivered by Equations (6)–(8) [27]:

(6) $$q_{e}=\frac{q_{\max}K_{L}C_{e}}{{1+K}_{L}C_{e}}$$

(7) $$q_{e}{=K}_{F}C_{e}{}^{\frac{1}{n}}$$

(8) $$q_{e}=\frac{RT}{b_{T}}\ln\left(a_{T}C_{e}\right)$$

where *q*_e_ (mg/g) represents the adsorption capacity at adsorption-desorption equilibrium; *q*_max_ (mg/g) is the maximized adsorption capacity predicted by Langmuir isotherm model; *K*_L_ (L/mg) is a Langmuir constant concerning the binding energy; *K*_F_ is a Freundlich constant and n is an indicator of adsorption intensity, *a*_T_ (L/g) and *b*_T_ (kJ/mol) are Temkin constants; *C*_e_ (mg/L) is remained Cr(VI) concentration at equilibrium; *T* (K) is temperature; and *R* is a gas constant (8.314 × 10^−3^ kJ/mol K).

## 3. Results and Discussion

### 3.1. Characterization

#### 3.1.1. SEM

**Figure 1:**
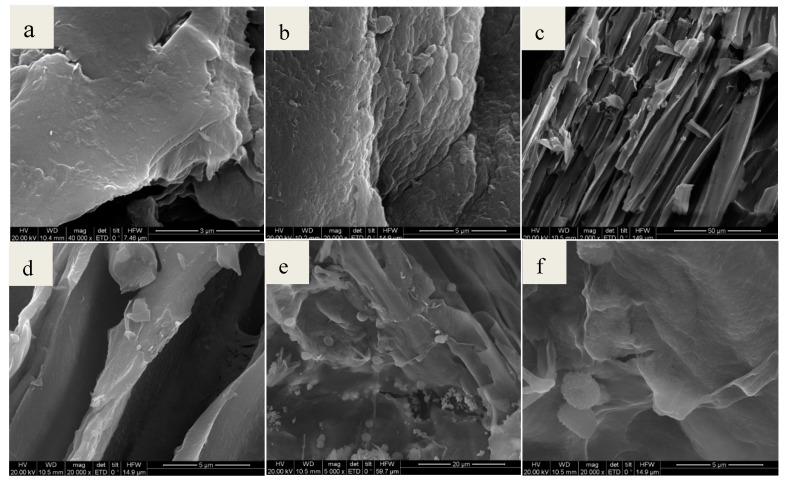
SEM images of (**a**) chitosan; (**b**) chitosan-BiFeO_3_; (**c**) and (**d**) chitosan-kenaf biochar; (**e**) and (**f**) CKB.

Scanning electron microscopy (SEM) was applied to observe the surface physical morphology of chitosan, chitosan-BiFeO_3_, chitosan-kenaf biochar, and CKB. As is seen from Figure 1a, chitosan has a rough surface texture and a multi-layered structure. Figure 1b shows some spherical particles of magnetic BiFeO_3_. The pore structure of chitosan-kenaf biochar is observed in Figure 1c,d because of the introduction of poly-porous kenaf biochar into chitosan, which facilitates Cr(VI) adsorption. Multi-layered chitosan, spherical BiFeO_3_, and porous kenaf biochar are integrated in SEM images of CKB (Figure 1e,f). CKB becomes smoother and it seems many tiny spherical particles are agglomerated on the surface of BiFeO_3_ nanoparticles as a result of interactions among chitosan, kenaf biochar and BiFeO_3_.

#### 3.1.2. XPS 

**Figure 2:**
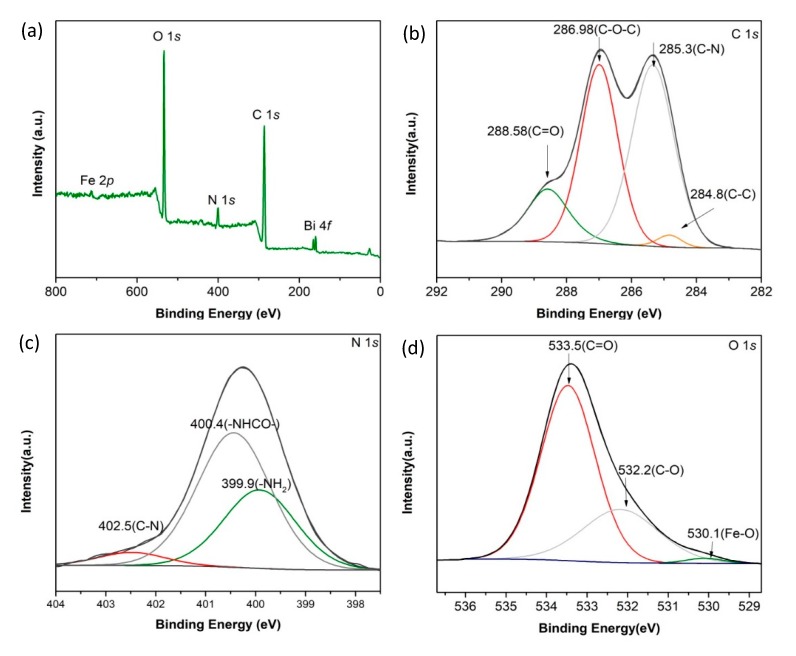
(**a**) XPS survey scan spectrum, (**b**) C 1s, (**c**) N 1s, and (**d**) O 1s XPS spectra of CKB.

The X-ray photoelectron spectroscopy (XPS) spectrum of CKB are shown in Figure 2. From Figure 2a, the peaks of Bi 4*f*, C 1*s*, N 1*s*, O 1*s*, and Fe 2*p* are observed at 158, 285, 400, 533, and 712 eV, respectively. The Bi 4*f* and Fe 2*p* peaks originate from BiFeO_3_ and the N1*s* peak largely comes from chitosan. From Figure 2b, the main peaks of the C 1*s* spectrum of CKB at binding energies of 284.8, 285.3, 286.98, and 288.58 eV can be assigned to C–C, C–N, C–O-C, and C=O groups [28], respectively, which belong to both chitosan and *kenaf* biochar. N 1*s* spectrum in Figure 2c shows three peaks at 399.9, 400.4, and 402.5 eV, referring to -NH_2_, -NHCO-, and C-N, respectively [29,30]. Figure 2d shows XPS peak deconvolution for O1*s* spectrum of CKB. Specifically, three peaks at 530.1, 532.2, and 533.5 eV are assigned the existence of Fe-O, C-O, and C=O groups, respectively [31]. These results suggest that *kenaf* biochar and BiFeO_3_ are successfully loaded on the crosslinked chitosan.

#### 3.1.3. EDS

**Figure 3:**
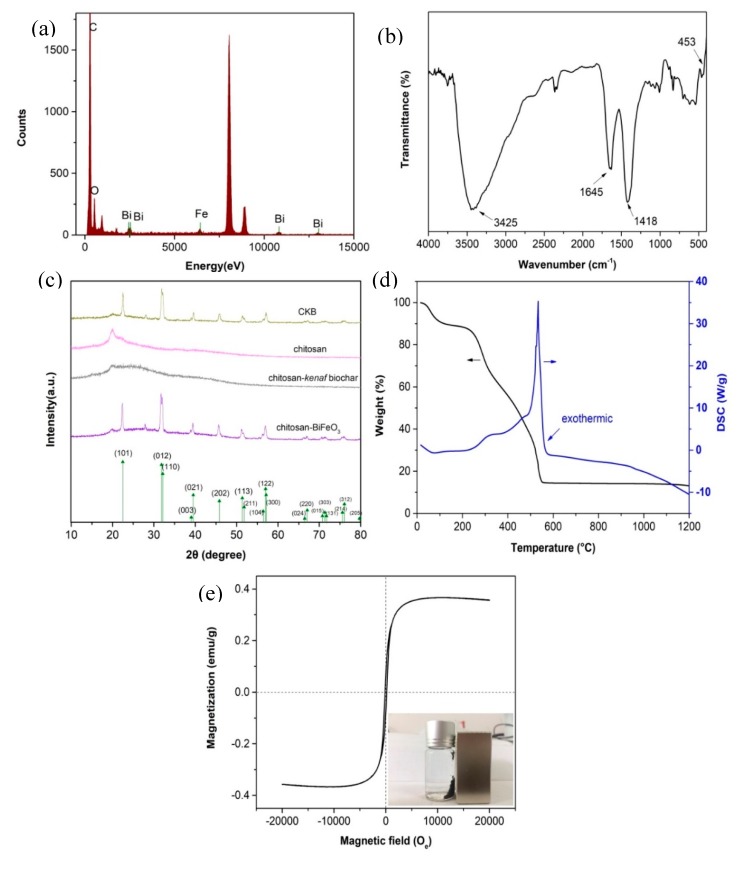
(**a**) EDS spectra of CKB, (**b**) FT-IR spectra of CKB, (**c**) XRD spectrum of chitosan, chitosan-BiFeO_3_, chitosan-kenaf biochar, and CKB, (**d**) TG-DSC of CKB, (**e**) magnetization curve of CKB.

Figure 3a shows the Energy Dispersive Spectroscopy (EDS) spectra for CKB, indicating the existence of element of Fe, Bi, O, and C. The elements of C come primarily from chitosan and *kenaf* biochar. The elements of Fe and Bi originate from BiFeO_3_ nanoparticles and the element of O may come from the oxygen-containing functional groups in BiFeO_3_ nanoparticles, *kenaf* biochar and chitosan. These results indicate that CKB is successfully prepared. 

#### 3.1.4. FT-IR

Fourier-transform infrared spectroscopy (FT-IR) of CKB is illustrated in Figure 3b. It shows four prominent peaks at 453, 1418, 1644, and 3425 cm^−1^. Specifically, the BiO_6_ octahedral structure in BiFeO_3_ lead to an adsorption peak at 453 cm^−1^ [32]. The adsorption observed at around 1418 cm^−1^ is attributed to C-N axial deformation in amino groups mainly from chitosan [31]. A peak at 1644 cm^−1^ represents C=O stretching in amide formed in glutaraldehyde crosslinking process of chitosan [17]. The wide and strong peak at around 3425 cm^−1^ refers to -OH and -NH_2_ stretching vibration [31]. These results confirm the successful fabrication of CKB. 

#### 3.1.5. XRD

Figure 3c shows X-ray diffraction (XRD) pattern of CKB, chitosan, chitosan-*kenaf* biochar, and chitosan-BiFeO_3_. Nine prominent characteristic peaks are observed in XRD patterns of CKB and chitosan-BiFeO_3_. The peaks are corresponding to major diffracted signals of the single-phase perovskite structure of BiFeO_3_ (JCPDS Card No. 20-169) [21], confirming that CKB and chitosan-BiFeO_3_ contain a certain amount of BiFeO_3_. Twin peaks are observed to merge together to form a particular peak, such as (012) and (110), (003) and (021), (113) and (211), (024) and (220), etc. Moreover, three peaks integrate together to form a single peak, such as (104), (122), and (300). These obvious peak-splitting indicate that the nanoparticles are rhombohedral [33]. XRD patterns of CKB and chitosan-BiFeO_3_ are similar because the synthesis does not change the crystal structure of BiFeO_3_. In addition, characteristic peaks of chitosan-*kenaf* biochar does not show in the XRD pattern of CKB, indicating that the carbon peaks tend to be overwhelmed by the strong diffraction of BiFeO_3_. 

#### 3.1.6. TG-DSC

The thermogravimetric and differential scanning calorimetry (TG-DSC) for CKB was conducted and its results were illustrated in Figure 3d. A slight weight loss (10%) is observed from 15 to 120 °C, suggesting a dehydration process. CKB loses little weight from 120 to 230 °C, but a sharp weight loss (75%) is observed when the temperature is higher than 230 °C, corresponding to decomposition of oxygen-containing functional groups such as epoxy, carboxyl, and hydroxyl groups in CKB and the pyrolysis of carbon skeleton. The stable weight of remains regardless of the rising temperature above 552 °C is ascribed to the mechanically stable amorphous iron oxide. It is noted that an exothermic peak at 532 °C observed in DSC curve indicates functional groups loss [34]. 

#### 3.1.7. VSM

The Vibrating Sample Magnetometer (VSM) of CKB in Figure 3e shows that the saturation magnetization (*M*_s_), coercivity (*H*_c_) and retentivity (*M*_r_) of the composite are 0.37 emu/g, 160 *O*_e_, and 0.067 emu/g, respectively. Near-zero retentivity and minor value of coercivity indicates the superparamagnetic property of CKB [35,36]. The inset in Figure 3e confirms that it can achieve solid-liquid separation quickly after adsorption via a magnet. 

### 3.2. Comparison Experiment

**Figure 4:**
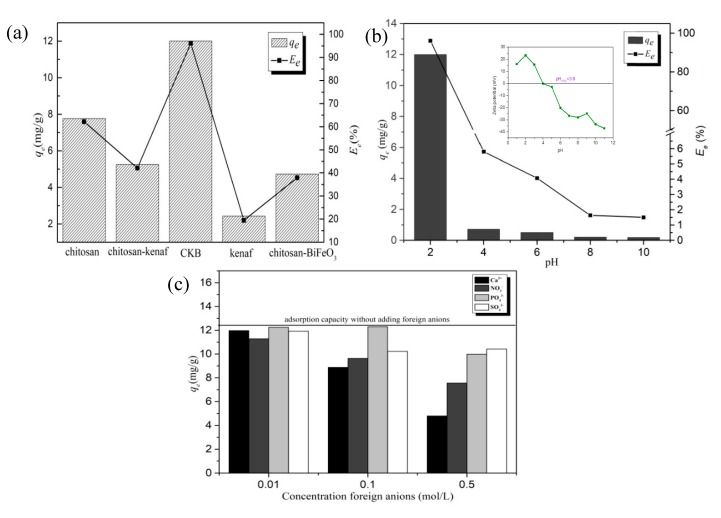
(**a**) Comparison of adsorption capacity among chitosan, chitosan-*kenaf* biochar, chitosan-BiFeO_3_, CKB and *kenaf* biochar: *C*_0Cr(VI)_ = 50mg/L, *W* = 0.2 g, *T* = 30 °C, *t* = 4 h, pH = 2, (**b**) Effect of solution pH on Cr(VI) adsorption onto CKB: *C*_0Cr(VI)_ = 50 mg/L, *W* = 0.2 g, *T* = 30 °C, *t* = 4 h, (**c**) Effect of Ca^2+^, NO_3_^−^, PO_4_^3−^, and SO_4_^2−^ on Cr(VI) adsorption onto CKB: *C*_0Cr(VI)_ = 50 mg/L, *W* = 0.2 g, *T* = 30 °C, *t* = 4 h, pH = 2.

Adsorption capacity 4g/L of chitosan, kenaf biochar chitosan-*kenaf* biochar, chitosan-BiFeO_3_, and CKB towards 50 mg/L of Cr(VI) for 4 h under pH 2.0 were examined, and results are recorded in Figure 4a. CKB, achieving the adsorption percentages towards Cr(VI) at 96.06%, respectively, possesses much higher adsorption efficiency than those of chitosan, kenaf biochar, chitosan-*kenaf* biochar, and chitosan-BiFeO_3_. It proves that CKB overcomes the setbacks of pure chitosan, *kenaf,* and BiFeO_3_ as adsorbents, exhibiting excellent efficiency of Cr(VI) decontamination. Reuse experiment of CKB for three cycles are shown in Figure S1.

### 3.3. Effect of pH

Solution pH is a decisive factor for adsorption in many relevant studies [6,20,37]. Figure 4b demonstrates that rise in pH value causes a dramatic decrease in adsorption capacity, which is similar as the results of the latest literatures [38] Specifically, adsorption percentage is 96.06% respectively when pH value is 2; yet, the figures descend sharply under 6% while it increases from 4 to 10. High dependence of adsorption efficiency on pH value can be ascribed to significant effects of pH value on surface charge of adsorbents, the degree of ionization, and the speciation of Cr(VI) species [39]. 

To estimate the magnitude of electronic charge on the surface of CKB, the zeta potentials of CKB under different pH environment were examined and the results are shown in the inset of Figure 4b. The pH_pzc_ value of CKB was measured to be 3.9. At pH lower than pH_pzc_, the surface of CKB was positively charged because of protonation of -NH_4_ groups on its surface, while CKB surface was negatively charged when pH > pH_pzc_ due to deprotonation of functional groups such as -NH_2_ and -COOH [40]. Cr(VI) exist mainly as HCrO_4_^−^ at low-pH environment, while CrO_4_^2−^ is a dominant form at alkaline solutions [41]. At pH < pH_zpc_, the removal of Cr(VI) was low, which was possibly due to an electrostatic attraction between positively charged CKB and negatively charged Cr(VI) ions (HCrO_4_^−^). It is notable that the highest positive zeta potential value (23.4 mV) was observed for CKB under pH 2.0, which was beneficial to the adsorption of Cr(VI) [42]. It explains why pH 2.0 is optimal for the uptake of Cr(VI) onto CKB. On the other hand, the electrostatic repulsion between both negatively charged CrO_4_^2−^ and CKB weakens Cr(VI) removal. In addition, OH^−^ may compete with CrO_4_^2−^ for adsorption sites, further reducing the adsorption capacity [37].

### 3.4. Effect of Foreign Anions

To investigate the effects of competing ions such as NO_3_^−^, SO_4_^2−^, PO_4_^3−^, and Ca^2+^, different concentration (0.01, 0.1, and 0.5 mol/L) of NaNO_3_, Na_2_SO_4_, Na_3_PO_4_, and CaCl_2_ were added into 50 mg/L of Cr (VI) solutions for reaction and the results are recorded in Figure 4c. The increasing amount of four kinds of ions reduces Cr(VI) adsorption; among them Ca^2+^ and NO_3_^−^ have the greatest inhibiting effect in all concentration gradients. One possible explanation is that CrO_4_^2−^ can react with Ca^2+^ to form CaCrO_4_, which is soluble at acidic solutions. CaCrO_4_ may have lower affinity to adsorption sites than Cr(VI) ions, thus reducing adsorption capacity. The inhibiting effects of inorganic anions NO_3_^−^, SO_4_^2−^, and PO_4_^3−^ can be ascribed to competition of these inorganic ions with Cr(VI) ions for adsorption sites, and NO_3_^−^ may be more easily attracted by sorption sites than SO_4_^2−^ and PO_4_^3−^ [43]. In addition, the fact that rises in ionic strength undermine the adsorption can imply that electrostatic attraction is probably a significant mechanism behind adsorption process of Cr(VI) onto the adsorbent [44,45]. To be clearer, positively charged adsorbent are surrounded by NO_3_^−^, SO_4_^2−^, and PO_4_^3−^, which forms an electrical diffused double layer, and high concentrations of inorganic ions can expand its thickness [45]. The thick layer extends the distance between the adsorbent and Cr(VI) ions, suppressing their electrostatic attraction, thus, the adsorption is reduced.

### 3.5. Adsorption Kinetics

**Figure 5:**
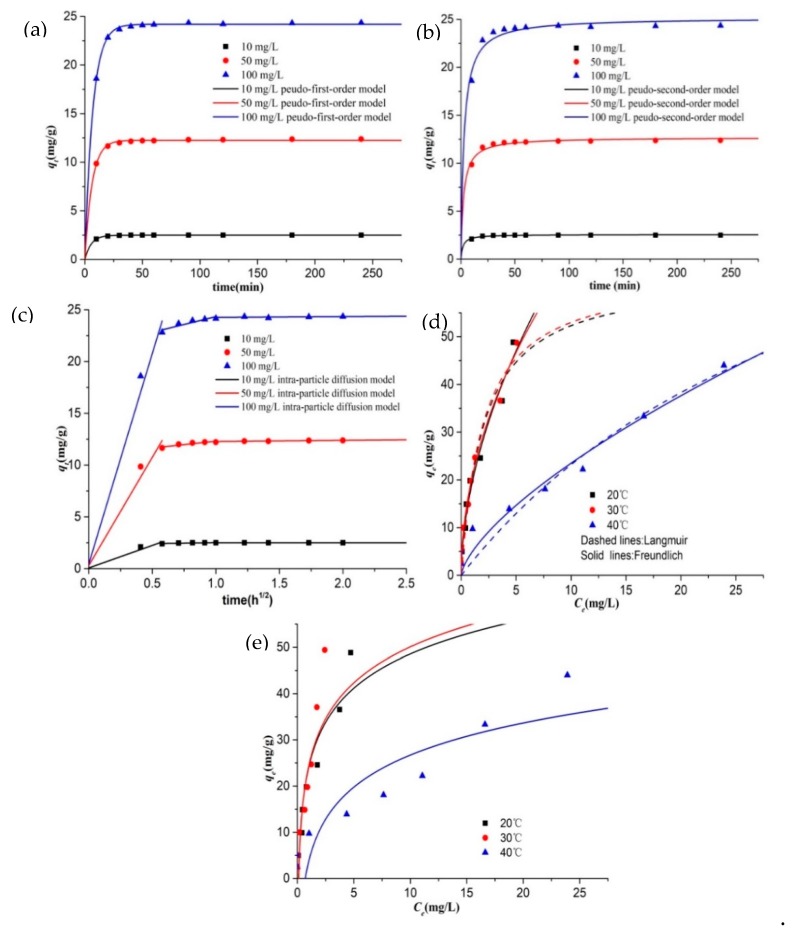
(**a**) Pseudo-first-order model, (**b**) Pseudo-second-order model, and (**c**) Intra-particle diffusion model of Cr(VI) adsorption at 10, 50, and 100 mg/L: *W* = 0.2 g, *T* = 30 °C, *t* = 4 h, pH = 2. (**d**) Langmuir and Freundlich isotherm model, (**e**) Temkin isotherm model of Cr(VI) adsorption onto CKB at 20, 30, and 40 °C, respectively, with the initial concentration of Cr(VI) at 10, 20, 40, 60, 80, 100, 150, and 200 mg/L: *W* = 0.2 g, *t* = 4 h, pH = 2.

Adsorption kinetics towards removal of different initial concentration (10, 50 and 100 mg/L) of Cr(VI) were explained by pseudo-first-order, pseudo-second-order, and intraparticle diffusion models in Figure 5a–c, and their parameters are summarized in Table 1. The adsorption reaches equilibrium after 90 min. Pseudo-first-order model can better explain the adsorption process over the whole contact time range as its correlation coefficient (*R*^2^) values are closer to 1 than those of pseudo-second-order model, and calculated equilibrated adsorption capacity (*q*_e,1_) for pseudo-first-order are closer to actual data. It indicated that chemisorption referring to sharing and exchange of electrons between sorbents and Cr(VI) ions is not the major rate-limiting step [23].

The adsorption process tends to contain three major steps: (1) external migration across the hydrodynamic boundary layer to surface of adsorbents; (2) intraparticle diffusion across liquid-filled pores into adsorbents and adsorption on sites; (3) reaching equilibrium [23,46,47]. As is seen from the multilinear *q*_t_ vs *t*^0.5^ curves in Figure 5c, adsorption of 10 mg/L of Cr(VI) does not experience the period of intraparticle diffusion. This is mainly because CKB contains sufficient adsorption sites on its surface provided for low level of Cr(VI) and only a few Cr(VI) ions diffuse across pores into CKB. However, apparently, film and intraparticle diffusion occur simultaneously when 50 and 100 mg/L of Cr(VI) are adsorbed and removed by CKB, and the intraparticle diffusion is not the only rate-limiting step [46]. 

### 3.6. Adsorption Isotherms

CKB was applied to remove different initial concentration of Cr(VI) at 10, 20, 40, 60, 80, 100, 150, and 200 mg/L under the temperature of 20, 30, and 40 °C, respectively. The obtained adsorption data was analyzed by Langmuir, Freundlich, and Temkin isotherm models and the results and isotherm parameters are, respectively, summarized in Figure 5d,e and Table 2. Apparently, the adsorption capacity rises consistently with rising initial concentration of Cr(VI), proving that CKB possesses sufficient adsorption sites that can meet the requirements of decontamination of high concentrations of Cr(VI). It is worth noting that the adsorption percentage maintains over 95% as initial concentration of Cr(VI) increases from 10 to 200 mg/L under the temperature of 30 °C, confirming impressive adsorption efficiency of CKB. The adsorption capacity for Cr(VI) at 20 and 30 °C are nearly the same, and the increased 10 °C slightly improves Cr(VI) adsorption. Nevertheless, the rise of temperature from 30 to 40 °C causes a sharp decrease in adsorption capacity. These results imply that the binding of Cr(VI) ions and CKB may be unstable at high temperature and the adsorption process may be exothermic [23,48]. Similar results were observed in chitosan-contained adsorbents, such as a study performed by Aydin and Aksoy utilizing chitosan flakes [48], a research by El-Reash using modified magnetic chitosan chelating resin [49], and the findings of Hassan Aslani applying magnetic chitosan and magnetic nanoparticles of chitosan modified with polyhexamethylene biguanide [50].

The Langmuir isotherm assumes that adsorption sites are homogenously dispersed on the adsorbent surface and one layer of metal ions independently cover the adsorbent surface [51]. The Freundlich model accounts for a non-ideal adsorption on a heterogeneous surface concerning physical and chemical interactions between adsorbates and adsorbents [51]. Temkin isotherm equation is based on the assumption that the heat of adsorption is linearly decreased with the coverage of molecules because of the repulsions between adsorbates and adsorbates [36,52]. The experimental data were greatly fitted with Langmuir model as the correlation coefficient (*R*^2^) values ranging from 0.923 to 0.945, suggesting that monolayer coverage may occur in the adsorption process. The value of *q*_max_ at 20, 30, and 40 °C are found to be 63.12, 63.57, and 109.50 mg/g, respectively. However, compared to Langmuir and Temkin model, the Freundlich model is more consistent with the adsorption data, with the *R*^2^ values between 0.95 and 0.968. It indicates that Cr(VI) ions are interacted with functional groups on the heterogeneous surface of the adsorbent. The Freundlich constants of n (1.819 for 20 °C, 1.979 for 30 °C, and 1.47 for 40 °C) are within desired adsorption range (1–10) [45], confirming effective adsorption ability of CKB. 

### 3.7. FFD for Investigating the Main and Mutual Effects of Multiple Factors

The experiment design matrix and its responses are illustrated in Table S1 and Figure S2 (Supplementary material). The main and simultaneous effects of multiple factors were investigated by a 2^5-1^ FFD and results are shown in Figure S3. Both of the runs have lowest pH values at 2 and highest initial concentration of Cr(VI) at 50 mg/L, confirming that low pH and high initial concentration of Cr(VI) promote the adsorption process.

The significance testing of FFD models were conducted. The half-normal plot in Figure S3a shows that factors A, C, and factor interaction AC have significant effects on the adsorption process. The pareto chart in Figure S3b further verifies high significance of these factors as t-value effects of factors A, C, and AC are above the Bonferroni limit. Based on these findings, a polynomial model predicting Cr(VI) adsorption can be expressed as Equation (9):

(9) $$q_{e}=\;-0.29625\;+\;0.21328\times \;A\;+\;0.26688\;\times \;C-0.024953\;\times \;\mathrm{AC}$$

To validify the FFD model obtained above, a normal probability plot of studentized residuals (Figure S3c) and a profile of the residuals as a function of predicted response values (Figure S3d) were conducted. Internally studentized residuals distribute along a straight line, indicating that regression residuals follow a normal pattern [53]. The predicted data versus actual values scatter randomly around a 45° line, confirming the predictive ability of the model [54]. Figure S3e shows that internally studentized residuals are in random scatter over the range of −3 to +3, confirming the adequacy of the FFD model.

The estimates of significant factors, and their mutual effects on Cr(VI) removal onto CKB, are shown in Figure S3f. The negative factors are A (−4.14) and D (−0.34), while ones with positive effects are C (13.37), B (1.27), and E (0.50). Their effect degree on Cr(VI) adsorption lie in the order C > A > B > E > D.

Typically, these selected factors have simultaneous effects on practical engineering [6]. Thus, the mutual effects of any two factors should be assessed. As is seen from Figure S4, parallel lines in cells AD, CD, and AE suggest that these factors influence adsorption independently [39]. The lines in cell AC show largest angle, indicating that solution pH (A) and initial concentration of Cr(VI) (C) could significantly affect each other. Overlapped lines in row D (NaCl) indicates the neglectable influence of NaCl on Cr(VI) removal [37].

**Figure 6:**
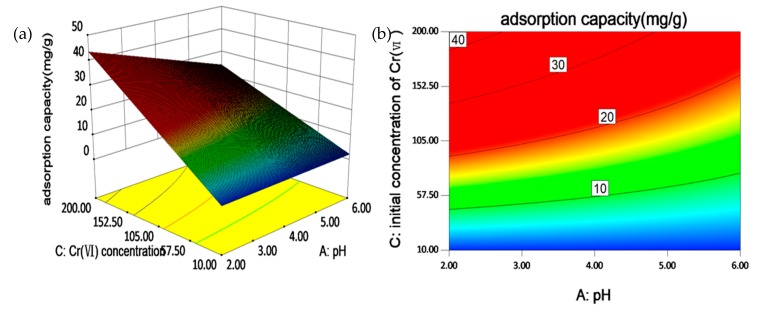
(**a**) Three-dimensional (3D) surface response plot and, (**b**) two-dimensional (2D) contour curves for AC (A: pH values; B: initial concentration of Cr(Ⅵ)) interactive effects on the adsorption of Cr(VI).

Figure 6 shows three-dimensional (3D) surface response plot and two-dimensional (2D) contour curves for AC interactive effects on the adsorption of Cr(VI). It can be clearly seen from the 3D surface response plot that high initial concentration and low pH simultaneously increase Cr(VI) adsorption. From the 2D contour curves, we find that at low pH, a slight rise of initial concentration Cr(VI) causes an obvious ascent of Cr(VI) adsorption capacity [55]. This indicates that high initial concentration of Cr(VI) is an essential factor for adsorption efficiency, and this effect is particularly promoted by low level of pH values [56].

### 3.8. Hypothesis of Cr(VI) Adsorption Mechanism onto CKB

To explore the mechanism of Cr(VI) adsorption onto CKB, the existing form of Cr onto CKB was analyzed by XPS and results were demonstrated in Figure S5. The peaks at binding energy of 576 and 578 eV are corresponding to Cr(III) and Cr(VI) of Cr 2*p*_3/2_ spectrum, respectively [46]. Therefore, both Cr(III) and Cr(VI) coexist onto CKB after adsorption. Analogous results of adsorption-coupled reduction were also reported [3,57]. The possible adsorption mechanism is as following: first, Cr(VI) ions are captured on the surface or inner pores of CKB through electrostatic attraction at acidic environment; second, some adsorbed Cr(VI) is reduced to Cr(III) under visible light, which is mainly because CKB contains a certain amount of BiFeO_3_ that exhibit the photocatalytic capacity and -NH_3_, -COOH, and other functional groups may also provide the electrons [3]; finally, Cr(III) is also adsorbed onto CKB, coexisting with Cr(VI). It is noted that during the adsorption process, Cr(III) may be oxidized back to Cr(VI) by photogenerated holes produced on CKB, and simultaneously, Cr(VI) ions are reduced to Cr(III). It will eventually reach an equilibrium.

## 4. Conclusions

A high-efficiency and reusable magnetic biochar-chitosan-BiFeO_3_ composite adsorbent (CKB) was successfully prepared and characterized in this study. The adsorption process of Cr(VI) onto CKB was evaluated under different pH, the existence of competing ions, various initial concentration of Cr(VI), and contact time. The results show that increase in pH values decreased the adsorption capacity of Cr(VI), and the optimum pH was 2. The adsorption capacity of CKB was much higher than chitosan, chitosan-*kenaf* biochar, and chitosan-BiFeO_3_ under the optimum pH, indicating that CKB compensate the disadvantages of the pure or binary materials as an adsorbent and yield much higher Cr(VI) decontamination efficiency. The presence of competing ions Ca^2+^, NO_3_^−^, SO_4_^2−^, and Cl^−^ weakened the adsorption of Cr(VI). Pseudo-first-order adsorption kinetics model and Freundlich-type adsorption isotherm could well describe the adsorption process, indicating a heterogeneous adsorption process referring to physical reaction of Cr(VI) ions and functional groups on the surface of CKB. The intra-particle diffusion model confirmed that the adsorption process included film diffusion, intra-particle diffusion and adsorption and equilibrium. In addition, a 2^5-1^ Fractional Factorial Design (FFD) indicate that pH values and initial concentration of Cr(VI) simultaneously affect the uptake of Cr(VI). Slightly increasing initial concentration of Cr(VI) in a low-pH environment could remarkably promoting the adsorption capacity of Cr(VI). The XPS analysis for Cr 2*p* spectrum on the surface of CKB after adsorption proves the ability of CKB to partially reduce Cr(VI) to low-toxicity Cr(III) by electrons generated by the photoactive BiFeO_3_ and the functional groups such as -NH_3_ and -COOH on its surface (suggested by FTIR and XPS analysis). In general, excellent adsorption efficiency of CKB indicates that it may be a good candidate for the remediation of Cr(VI)-contaminating wastewater.

## Figures and Tables

**Table 1:** Adsorption kinetics parameters for Cr(VI) adsorption onto CKB.

Model	Parameters	Initial Concentration of Cr(VI) (mg/L)		
		10	50	100
Peudo-first-order model	*q*_e,1_	2.497	12.257	24.19
	*K*_2_	0.179	0.16	0.145
	*R* ^2^	0.996	0.981	0.993
Peudo-second-order model	*q*_e,2_	2.567	12.701	25.178
	*K*_2_	0.209	0.032	0.013
	*R* ^2^	0.841	0.909	0.879

**Table 2:** Adsorption isotherm parameters for Cr(VI) adsorption onto CKB.

Models	Parameters	Temperature (°C)		
		20	30	40
Langmuir	*q*_max_	63.116	63.566	109.497
	*K*_L_	0.484	0.502	0.027
	*R* ^2^	0.94	0.945	0.923
Freundlich	*n*	1.819	1.979	1.47
	*K*_F_	19.442	20.72	4.91
	*R* ^2^	0.967	0.968	0.95
Temkin	*a*_T_	9.494	8.745	1.44
	*b*_T_	0.228	0.224	0.26
	*R* ^2^	0.895	0.908	0.815

## Supplementary Materials

The following are available online at https://www.mdpi.com/1660-4601/17/3/788/s1. Table S1. Experimental design matrix of the 2^5−1^ FFD for Cr(VI) adsorption onto CKB. Figure S1. Reuse experiment of CKB for three cycles. Figure S2. Cr(VI) removal onto CKB under various experimental conditions designed by 2^5-1^ FFD. Figure S3. Identification of main factors and interaction factors on Cr(VI) adsorption by CKB. Figure S4. Interaction effects plot for Cr(VI) decontamination. Figure S5. Cr 2p XPS spectra of CKB after adsorption.
