# Supplementary material for: Coupling of kenaf Biochar and Magnetic BiFeO3 onto Cross-Linked Chitosan for Enhancing Separation Performance and Cr(VI) Ions Removal Efficiency
Source: Int J Environ Res Public Health. 2020 Jan 27;17(3):788. doi: 10.3390/ijerph17030788 (PMC7037466; doi:10.3390/ijerph17030788)
Supplement: Supplementary file 1 [file ijerph-17-00788-s001.pdf]

International Journal of *Environmental Research and Public Health*

MDPI

Supplementary Material for

Coupling of *kenaf* biochar and magnetic $BiFeO_3$ onto cross-linked chitosan for enhancing separation performance and Cr(VI) ions removal efficiency

Daixi Zhou[1], Guangyu Xie[1], Xinjiang Hu[1,*], Xiaoxi Cai[2], Yunlin Zhao[4], Xi Hu[1,*], Qi Jin[4], Xiaohua Fu[1], Xiaofei Tan[3], Chong Liang[1], Kaiqi Lai[1], Hui Wang[1], Chunfang Tang[1]

[1] College of Environmental Science and Engineering, Central South University of Forestry and Technology, Changsha 410004, P.R. China; daixizhou07@126.com (D.Z.); anyuxie@126.com(G.X.); xjhu@csuft.edu.cn(X.J.H.); T20152243@csuft.edu.cn(X.H.); T20031513@csuft.edu.cn (X.F.); 15964519539@163.com(C.L.); lkq1799689599@163.com(K.L.); wanghui@csuft.edu.cn(H.W.); T20051104@csuft.edu.cn (C.T.)

[2] College of art and design, Hunan First Normal University, Changsha 410205, P.R. China; xiaoxi@hnu.edu.cn (X.C.)

[3] College of Environmental Science and Engineering, Hunan University, Changsha 410082, P.R. China; tanxf@hnu.edu.cn(X.T.)

[4] Faculty of Life Science and Technology, Central South University of Forestry and Technology, Changsha 410004, P.R. China; T20142201@csuft.edu.cn（Y.Z.）; jq305745239@126.com(Q.J)

[*] Correspondence: huxinjiang@126.com, xjhu@csuft.edu.cn (Xinjiang Hu); T20152243@csuft.edu.cn (Xi Hu)

Table S1 Experimental design matrix of the 2^{5-1} FFD for Cr(VI) adsorption onto CKB. (A: pH, B: temperature, C: initial concentration of Cr(VI), D: NaCl, E: KH_2PO_4)

Run number	Values of independent variables*				
	A	B	C	D	E
1	2	50	90	0.1	0.01
2	2	10	90	0.1	0.1
3	6	50	90	0.01	0.01
4	6	50	90	0.1	0.1
5	6	10	90	0.01	0.1
6	2	10	90	0.01	0.01
7	2	50	90	0.01	0.1
8	6	10	90	0.1	0.01
9	2	10	10	0.01	0.1
10	6	10	10	0.01	0.01
11	6	50	10	0.01	0.1
12	2	50	10	0.1	0.1
13	2	50	10	0.01	0.01
14	6	10	10	0.1	0.1
15	2	10	10	0.1	0.01
16	6	50	10	0.1	0.01

The regeneration ability of an adsorbent is considered as a significant factor to assess its cost-effectiveness [1] [2]. In order to investigate the regenerability and reusability of CKB, the adsorption-desorption cycle experiments were performed on CKB. At first, 12 mg/g of Cr(Ⅵ) was loaded onto CKB by contacting 0.2 g CKB with 50 mg/L of Cr(Ⅵ) solution (50 mL) at pH 2.0 in a thermostatic shaker at 150 rpm under 30 ℃. Then for desorption, Cr(Ⅵ)-loaded CKB was separated from solutions using a magnet and then washed by ultrapure water for three times. Next, Cr(Ⅵ)-loaded CKB was contacted with 50 mL of 0.5 M NaOH in a thermostatic shaker at 150 rpm under 30 ℃. After washing CKB by ultrapure water for three times, the adsorbent was reused in the next adsorption cycle. Three consecutive adsorption-desorption cycles were examined.

As shown in the first two cycles in Fig. S1, the adsorption capacity of CKB was 12 mg/g. In the third cycle the adsorption capacity of CKB was slightly reduced to 9 mg/g. Therefore, CKB can be successfully reused for two adsorption-desorption cycles without loss of its original adsorption capacity.

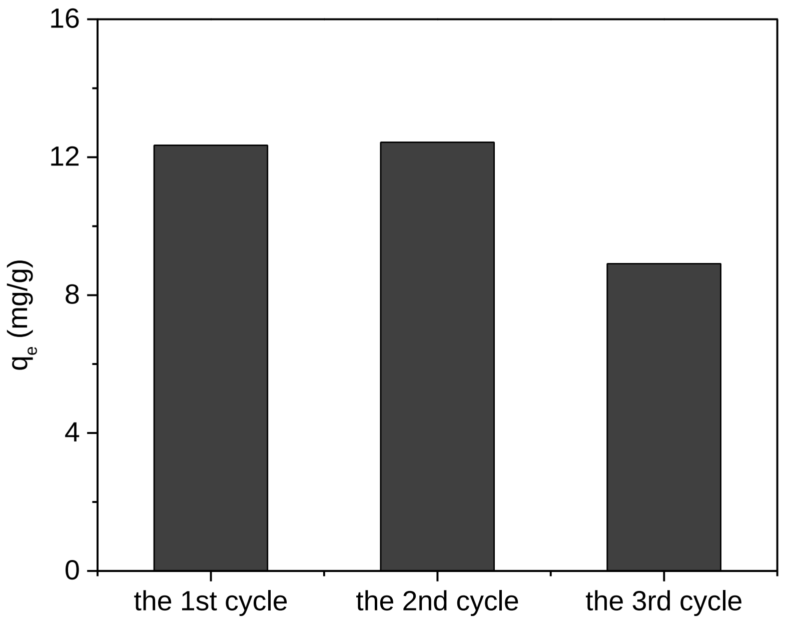

Figure S1. Reuse experiment of CKB for three cycles: $C_{0Cr(VI)}$ = 50mg/L, W = 0.2 g, T = 30 °C, t = 4 h, pH = 2.

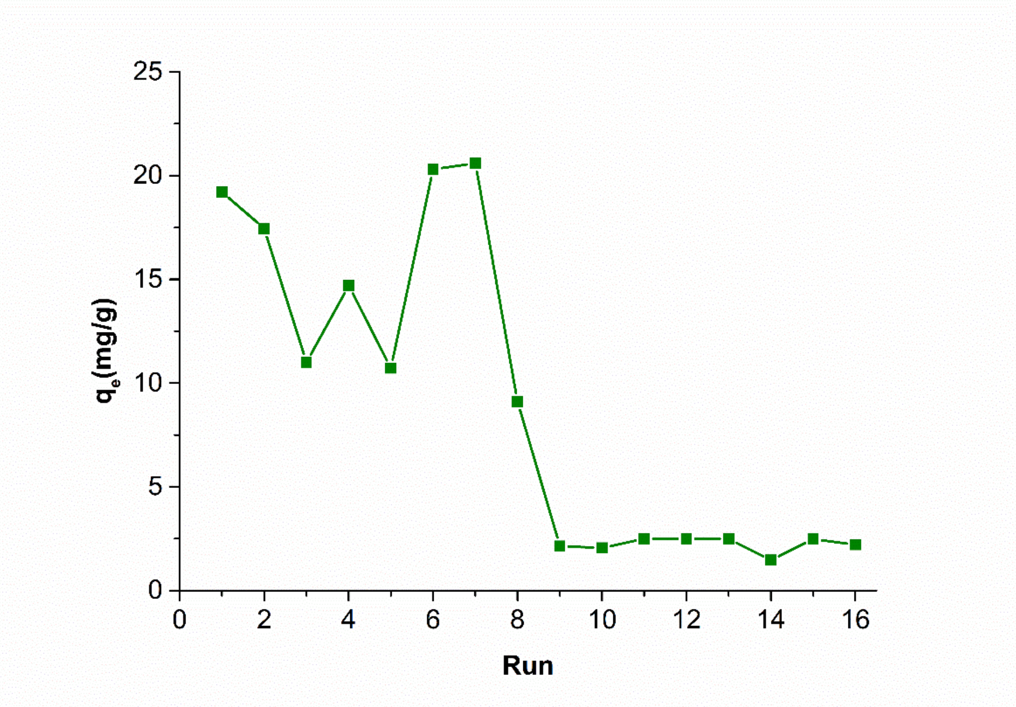

Figure S2 Cr(VI) removal onto CKB under various experimental conditions designed by 2^{5-1} FFD

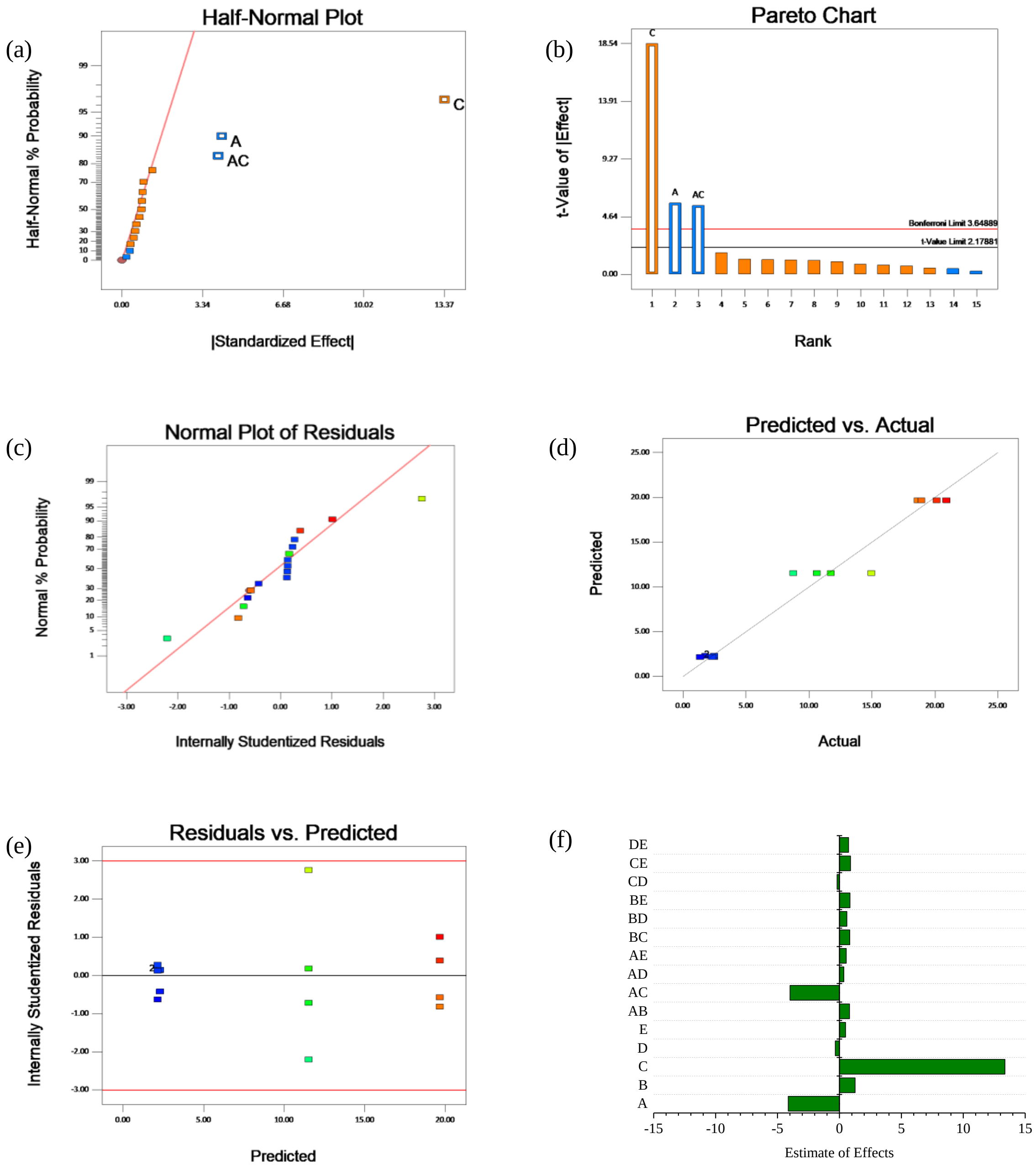

Figure S3 (a) Half-normal probability plot; (b) Pareto chart; (c) Normal plot of residuals; (d) Comparison of predicted and experimental adsorption capacities; (e) Plots of internally standardized residuals with predicted values; (f) Estimates of effects: Identification of main factors and interaction factors on Cr(VI) adsorption by CKB: (A) pH; (B) Temperature; (C) initial concentration of Cr(VI); (D) NaCl; (E) KH_2PO_4.

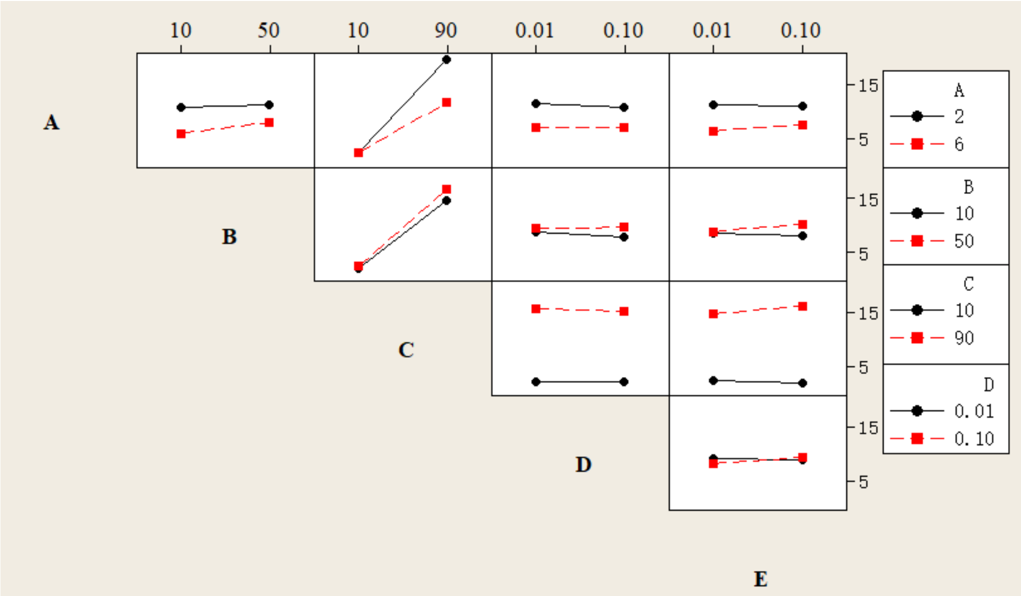

Figure S4 Interaction effects plot for Cr(VI) decontamination: (A) pH; (B) Temperature; (C) initial concentration of Cr(VI); (D) NaCl; (E) KH_2PO_4.

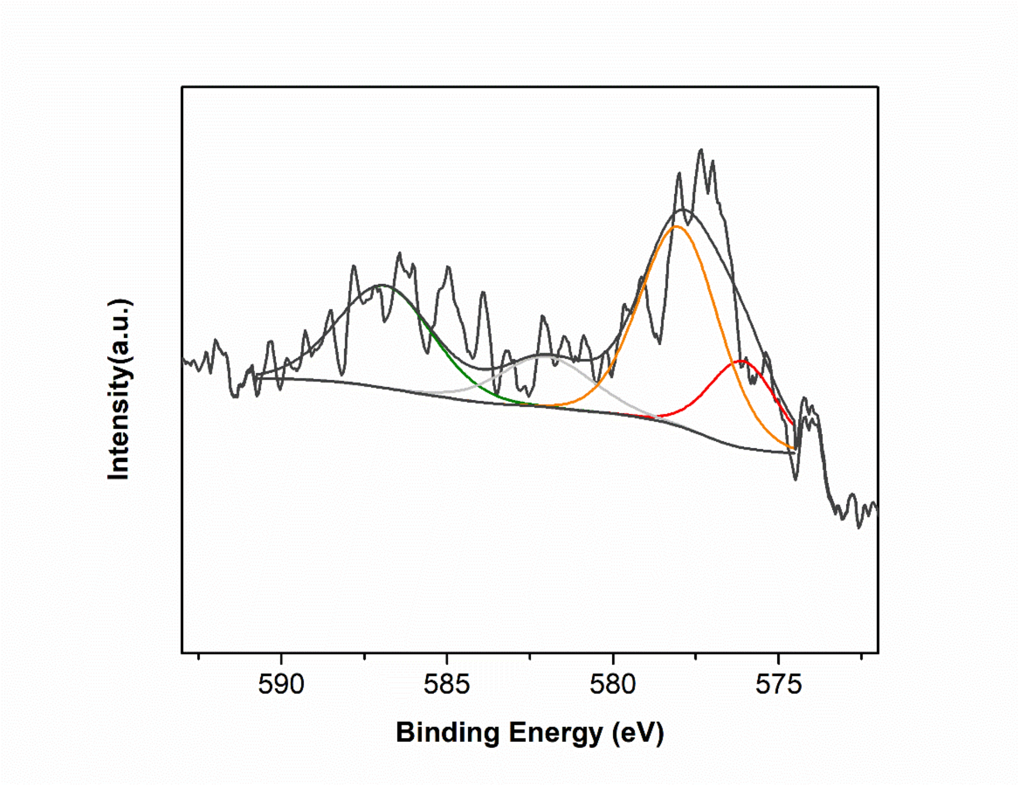

Figure. S5 Cr 2*p* XPS spectra of CKB after adsorption.

References

1. Bhaumik, M.; Maity, A.; Srinivasu, V.V.; Onyango, M.S. Enhanced removal of Cr(VI) from aqueous solution using polypyrrole/Fe_3O_4 magnetic nanocomposite. *Journal of hazardous materials* **2011**, *190*, 381-390, doi:10.1016/j.jhazmat.2011.03.062.
2. Vilardi, G.; Ochando-Pulido, J.M.; Stoller, M.; Verdone, N.; Di Palma, L. Fenton oxidation and chromium recovery from tannery wastewater by means of iron-based coated biomass as heterogeneous catalyst in fixed-bed columns. *Chemical Engineering Journal* **2018**, *351*, 1-11, doi:10.1016/j.cej.2018.06.095.
